# Quantification of Intracranial Structures Volume in Fetuses Using 3-D Volumetric MRI: Normal Values at 19 to 37 Weeks' Gestation

**DOI:** 10.3389/fnins.2022.886083

**Published:** 2022-05-12

**Authors:** Jing-Ya Ren, Ming Zhu, Guanghai Wang, Yiding Gui, Fan Jiang, Su-Zhen Dong

**Affiliations:** ^1^Department of Radiology, Shanghai Children's Medical Center, School of Medicine, Shanghai Jiao Tong University, Shanghai, China; ^2^Pediatric Translational Medicine Institution, Shanghai Children's Medical Center, School of Medicine, Shanghai Jiao Tong University, Shanghai, China; ^3^Department of Developmental and Behavioral Pediatrics, Shanghai Children's Medical Center, School of Medicine, Shanghai Jiao Tong University, Shanghai, China; ^4^MOE-Shanghai Key Laboratory of Children's Environmental Health, School of Medicine, Xinhua Hospital, Shanghai Jiao Tong University, Shanghai, China; ^5^Shanghai Center for Brain Science and Brain-Inspired Technology, Shanghai, China

**Keywords:** fetal brain development, magnetic resonance imaging, three dimensional volumetric, prenatal diagnosis, image processing

## Abstract

**Objective:**

The purpose of this study is to establish a reference of intracranial structure volumes in normal fetuses ranging from 19 to 37 weeks' gestation (mean 27 weeks).

**Materials and Methods:**

A retrospective analysis of 188 MRI examinations (1.5 T) of fetuses with a normal brain appearance (19–37 gestational weeks) from January 2018 to December 2021 was included in this study. Three dimensional (3-D) volumetric parameters from slice-to-volume reconstructed (SVR) images, such as total brain volume (TBV), cortical gray matter volume (GMV), subcortical brain tissue volume (SBV), intracranial cavity volume (ICV), lateral ventricles volume (VV), cerebellum volume (CBV), brainstem volume (BM), and extra-cerebrospinal fluid volume (e-CSFV), were quantified by manual segmentation from two experts. The mean, SD, minimum, maximum, median, and 25th and 75th quartiles for intracranial structures volume were calculated per gestational week. A linear regression analysis was used to determine the gestational weekly age-related change adjusted for sex. A *t*-test was used to compare the mean TBV and ICV values to previously reported values at each gestational week. The formulas to calculate intracranial structures volume derived from our data were created using a regression model. In addition, we compared the predicted mean TBV values derived by our formula with the expected mean TBV predicted by the previously reported Jarvis' formula at each time point. For intracranial volumes, the intraclass correlation coefficient (ICC) was calculated to convey association within and between observers.

**Results:**

The intracranial volume data are shown in graphs and tabular summaries. The male fetuses had significantly larger VV compared with female fetuses (*p* = 0.01). Measured mean ICV values at 19 weeks are significantly different from those published in the literature (*p* < 0.05). Means were compared with the expected TBV generated by the previously reported formula, showing statistically differences at 22, 26, 29, and 30 weeks' gestational age (GA) (all *p* < 0.05). A comparison between our data-derived formula and the previously reported formula for TBV showed very similar values at every GA. The predicted TBV means derived from the previously reported formula were all within the 95% confidence interval (*CI*) of the predicted means of this study. Intra- and inter-observer agreement was high, with an intraclass correlation coefficient larger than 0.98.

**Conclusion:**

We have shown that the intracranial structural volume of the fetal brain can be reliably quantified using 3-D volumetric MRI with a high degree of reproducibility and reinforces the existing data with more robust data in the earlier second and third stages of pregnancy.

## Introduction

Currently, ultrasound (US) biometry is the reference standard for assessing fetal brain development (Griffiths et al., 2017). With regard to the central nervous system (CNS), indirect indicators of fetal brain development are used routinely by measurement of two-dimensional (2-D) parameters (De Oliveira Júnior et al., 2021), such as biparietal diameter (BPD) and head circumference (HC) (Ruiz et al., 2017; Kline-Fath, 2019; Sibbald et al., 2021). However, BPD and HC can only be compared with the size of the head, including the skull, and the sizes of the brain and detailed study of different intracranial structures cannot be performed (Fried et al., 2021).

Although US has been the primary imaging method for prenatal screening for fetal brain anomalies, fetal MRI has become a useful supplemental imaging tool. Fetal MRI has been useful in evaluating abnormalities of fetal structures which are difficult to thoroughly evaluate by prenatal US alone, with obvious advantages over US in displaying neurological maturation and abnormalities (Grossman et al., 2006; Ruiz et al., 2017). The use of three-dimensional (3-D) volumetric *in utero* MRI is a relatively newer modality and allows more accurate measurement of intracranial structure volumes, which can more accurately reflect the growth of the fetal brain than 2-D parameters (Blondiaux and Garel, 2013; Jarvis and Griffiths, 2017; Kyriakopoulou et al., 2017; Takakuwa et al., 2021).

Although several existing studies (Clouchoux et al., 2012; Griffiths et al., 2019) have attempted to establish the normative MR data for intracranial compartment volume at varying gestational ages (GAs) as measured by fetal MRI, however, these have some limitations. Most studies had a relatively small GA range (Corbett-Detig et al., 2011; Clouchoux et al., 2012), as well as limited measurements of regional brain structures (Andescavage et al., 2017), thicker slice thickness of MRI scans (Gholipour et al., 2011). In our study, we aim to provide reference values for normal fetal intracranial structure volumes to reinforce existing data with more reliable normative data in the second and third stages of pregnancy. This is essential to understand the progression and timing of aberrant brain development and early detection of deviations from normal growth during this period.

## Materials and Methods

### Subjects

A retrospective study at our institution was performed. Fetal brain MRI databases spanning the years 2018–2021 in our medical center were searched for examinations performed between 18 and 38 weeks of gestation (median GA: 27 weeks). These data were from pregnant women who had been acquainted with the procedure and possible risks of fetal MRI and had given written informed consent to conduct prenatal studies before the examination. This study protocol was authorized by the review board of our medical hospital. All methods of the study were performed in accordance with the relevant guidelines and regulations.

We created a normative database by scanning low-risk pregnant women who were enrolled in our control cohort group. Inclusion criteria: women who had a previous child with a confirmed abnormality or US query of non-CNS mild abnormalities without brain abnormalities seen on fetal MRI. Fetal age was based on the first day of the last normal menstrual period and confirmed by a first-trimester US scan.

Exclusion criteria: twin or multiple pregnancies, fetal malformation or chromosomal abnormalities, associated arrhythmias, perinatal infections, fetal anemia, and maternal conditions that may affect fetal hemodynamics, such as thyroid disease, pregestational diabetes, and pre-eclampsia. The malformation of non-CNS that may be affect CNS development can also be excluded. Excessive fetal motion artifact prevents the acquisition of three orthogonal planes for reconstruction and measurement.

### MRI Protocol and Analysis

All fetal MRI scans were performed using a Philips Achieva 1.5 T MRI scanner and a 16-channel sense-xl-torso coil (Philips Healthcare). Pregnant women were in the supine or the left-sided position. No maternal or fetal sedation was used during the MR imaging examinations. First, localizer images were acquired to determine the location of the fetal head. The following parameters were used for the single-shot fast spin-echo (SSFSE) sequence: TR/TE: 12,000/80 ms, matrix: 236 × 220, flip angle: 90 degrees, field of view: 260–355 mm^2^, and slice thickness: 2 mm with 0-mm spacing. The scan time of SSFSE sequence was 15–45 s. The repeat data acquisition or breath-holding of pregnant women at the end of expiration or both was used to reduce motion artifacts to improve the success of the SSTSE sequence.

### MRI Processing and Segmentation

**Figure 1:**
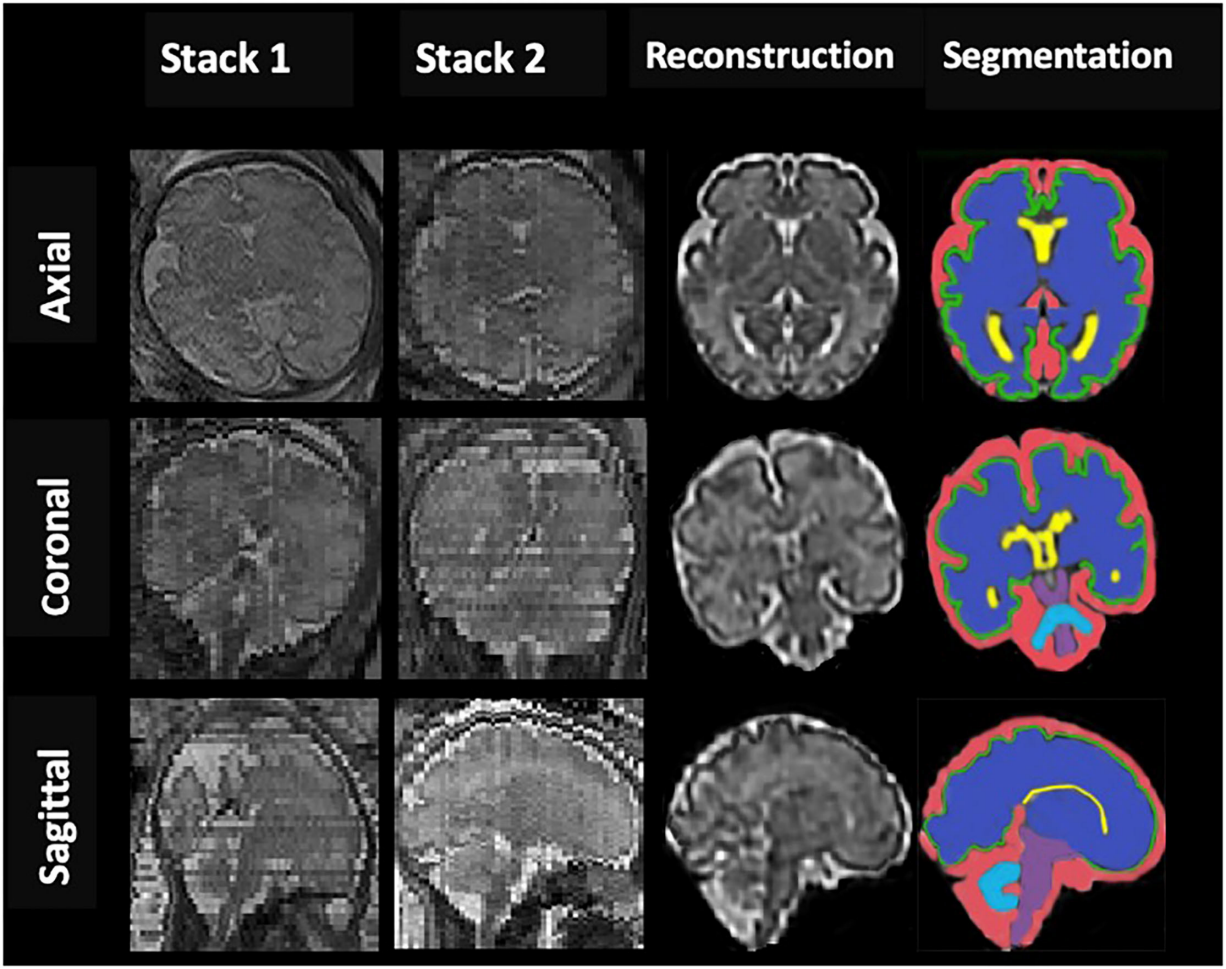
An example of fetal brain reconstruction from two dimensional (2D) single-shot fast spin-echo (SSFSE) MRI slices of axial, sagittal, and coronal planes (Stack1; 2) to a single three dimensional (3D) reconstruction volumetric image (reconstruction column); 3D reconstructed brain of a normal control fetus at 32 gestational weeks with manual 3D segmentations of supratentorial brain tissue, lateral ventricles, cortex, cerebellum, brainstem (BM), and extra-cerebrospinal fluid volume (eCSF) (segmentation column).

The post-acquisition processing was performed using the Linux workstation. The acquired data were converted from DICOM (Digital Imaging and Communications in Medicine) to NIfTI (Neuroimaging Informatics Technology Initiative) format with MATLAB (The MathWorks Inc, Natik, MA) and DCM2NII software (version 12.12.2012). For each subject, a single 3D motion-corrected high-resolution brain volume was reconstructed from the 2D SSTSE imaging stacks using a slice-to-volume reconstruction (SVR) method (Jiang et al., 2007; Gholipour et al., 2011). First, we used an atlas-based method to extract a mask of the brain by defining a region of interest (ROI) from surrounding fetal and maternal tissue in each of the 3 principal planes, namely, sagittal, coronal, and axial. Second, images were processed using the non-parametric non-uniform intensity normalization algorithm to correct for intensity inhomogeneity to get a consistent, spatially invariant, signal intensity distribution for each brain tissue. After that, the high-resolution isotropic reconstructed 3D volumetric images with the resolution of 0.5 × 0.5 × 0.5 mm were reconstructed from the registered low resolution and motion-corrupted 2D slices by using the Gauss–Seidel and super-resolution reconstruction method (Gholipour et al., 2010; Askin Incebacak et al., 2022) (Figure 1).

Coronal slices were segmented manually by editing using ITK-SNAP software (version3.8, http://www.itksnap.org/) to volumetric measure in intracranial cavity volume (ICV), total brain volume (TBV), lateral ventricles volume (VV), and extra-cerebrospinal fluid (e-CSFV). Besides, based on the created label-maps, gray matter (GMV), subcortical brain (SBV), cerebellar (CBV), and brainstem (BM) volumes were determined, and total brain (TBV = GMV + SBV+CBV+BM) and intracranial cavity (ICV=TBV+VV+ e-CSFV) volumes were calculated. All intracranial CSF spaces surrounding the supratentorial brain structures and infratentorial regions were included in E-CSF but not any ventricular tissue. Lateral ventricles volume represented the volume of left and right lateral ventricles. Volumes were determined by multiplying the voxel count by the number of voxels in the segmentation and converting to cubic centimeters (Figure 1). Raters were blinded to the patient's identity and gestation for all subjects.

The relative growth rate represents the percentage volume gain relative to the average volume for each intracranial structure and was calculated using the formula (Hoffmann and Poorter, 2002): [(lnV2 – lnV1)/(GA2 – GA1)] × 100, where ln is the natural logarithm, GA1and GA2 are the gestational weeks at a given GA range, and V1 and V2 are the average volumes of different intracranial structures corresponding to GA1 and GA2 at the time point, respectively.

### Statistical Analysis

Statistical analysis was performed using SPSS 22.0 software. The mean, SD, minimum, maximum, median, and 25th and 75th quartiles were calculated for the measured volumes of TBV, GMV, SBV, ICV, e-CSFV, VV, CBV, and BM at each GA and presented in tabulated form. A *t*-test was used to compare our mean TBV and ICV to previously reported values (Jarvis et al., 2016, 2019). Scatter plots were drawn according to the segmented volumes against GA and adjusted for sex, then a quadratic line showed the best fit for TBV, GMV, SBV, ICV, e-CSFV, VV, CBV, and BM with 95% confidence intervals (*CI*s). Then, the new formulas to calculate intracranial structures derived from our data were created. Jarvis' formula for calculating fetal TBV was derived from a fitting standard curve (TBV = 0.53 GA^2^ – 13.33 GA + 89.69 [R^2^ = 0.97]). Subsequently, student's *t*-tests analysis was performed to compare the predicted mean TBV values at each GA derived by our formula with the expected mean TBV predicted by the Jarvis' formula. About 30% of scans were randomly selected and were corrected by the same observer and another observer. For intracranial volumes, the intraclass correlation coefficient (ICC) was calculated to convey association within and between observers. The values of *p* were considered statistically significant when <0.05.

## Results

**Figure 2:**
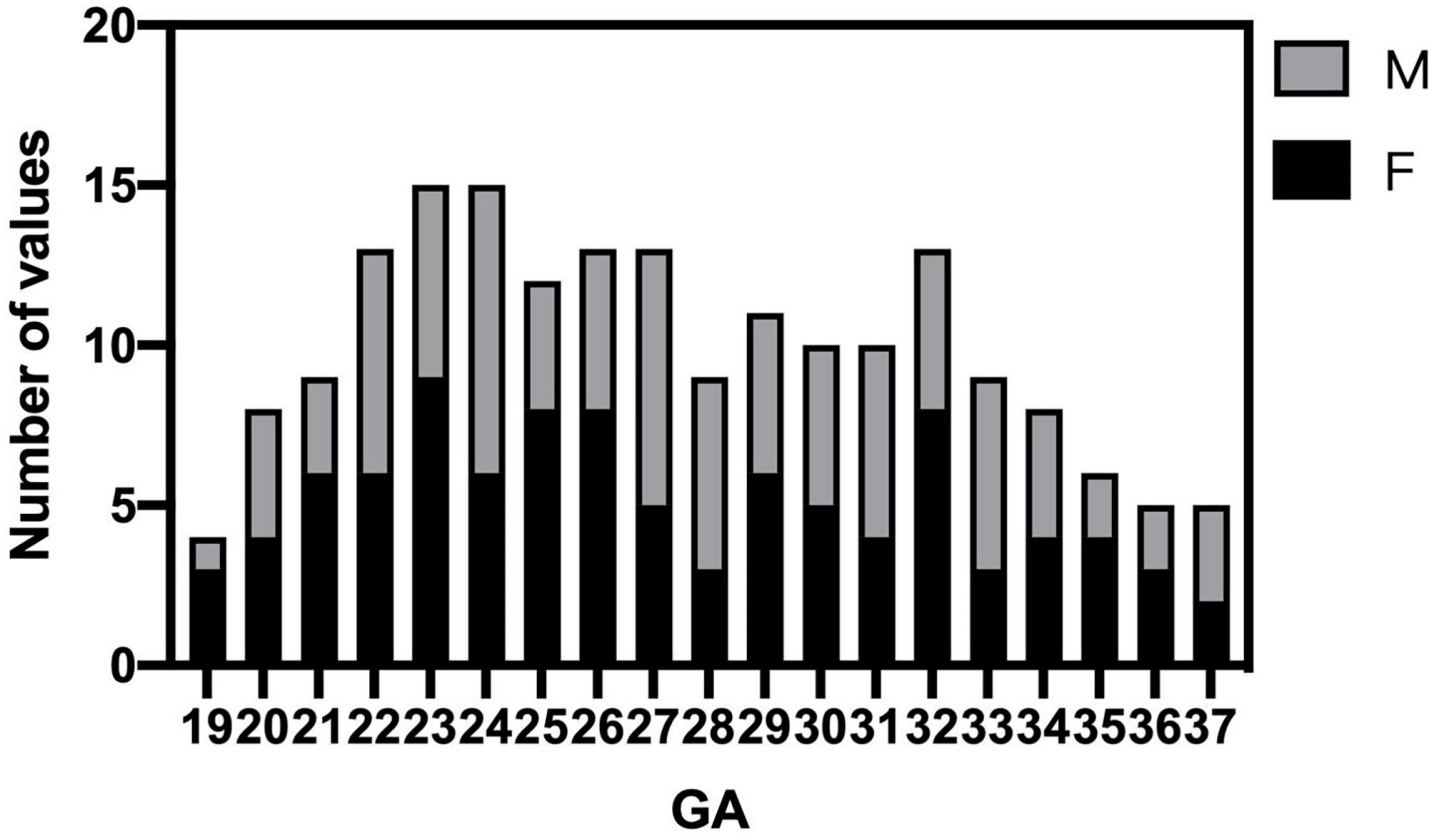
Histogram of gestational age (GA) and sex distribution of MR scans in normal fetuses (*n* = 188).

Fetal brain MRI data were collected from 700 singleton pregnancy fetuses at a GA between 18 and 38 weeks. After excluding 512 normal fetal brain data with noticeable motion artifacts that resulted in low-quality data and gross errors in segmentation, a total of 188 normal fetal brains (97 female fetuses/91 male fetuses) were analyzed between 19 and 37 GA. The GA of the fetuses ranged from 19 to 37 weeks (mean, 27.4 ± 4.8 weeks) is shown in Figure 2. Intra- and inter-observer agreement for supratentorial brain structures and infratentorial regions was high, with ICCs all larger than 0.98.

**Figure 3:**
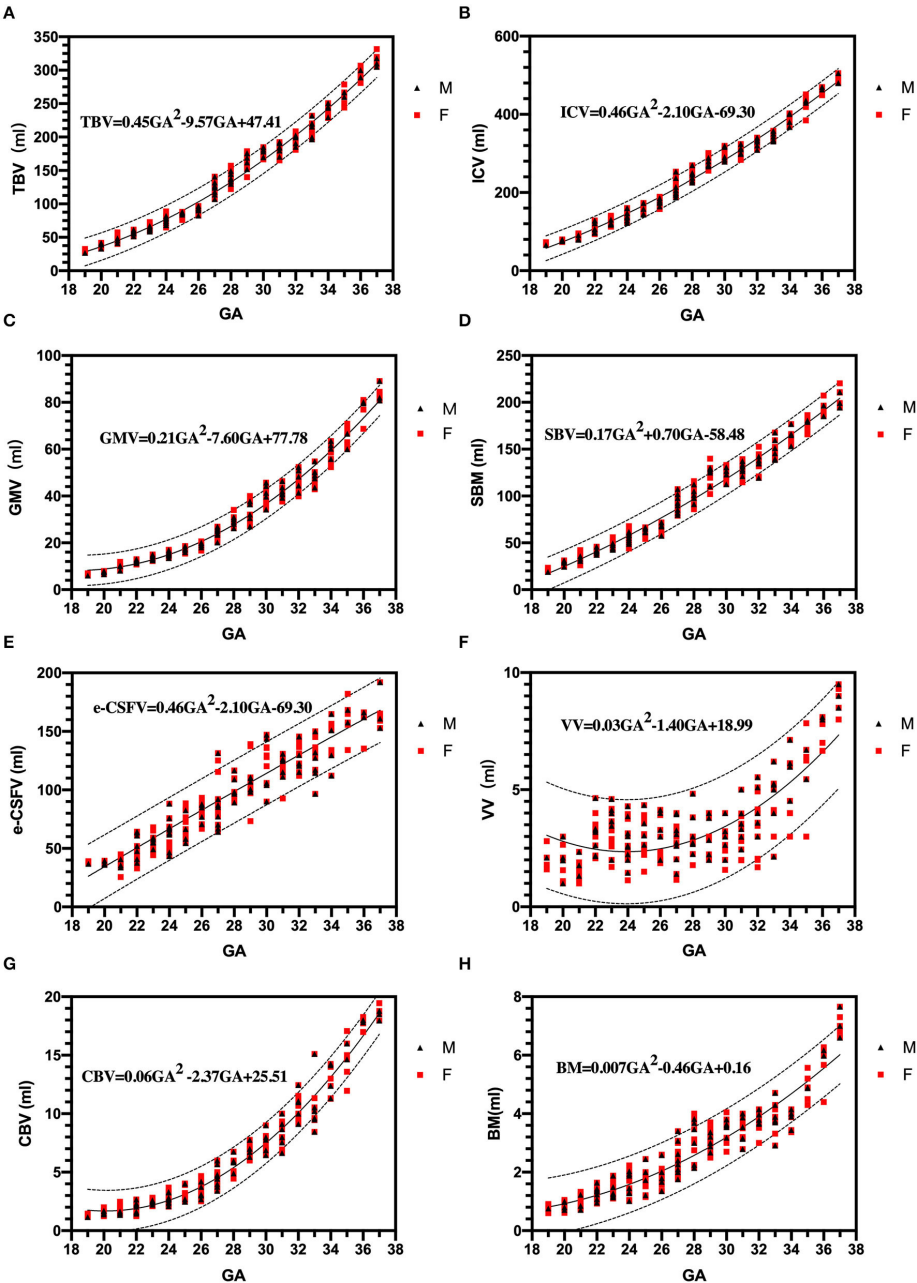
Three dimensional measurements: growth trajectories and centiles. Best fit models for normal control 3D growth trajectories of an intracranial brain tissue, **(A)**: brain volume (TBV), **(B)**: intracranial cavity volume (ICV), **(C)**: gray matter volume (GMV), **(D)**: subcortical brain volume (SBV), **(E)**: extra-cerebrospinal fluid (e-CSFV), **(F)**: lateral ventricles volume (VV), **(G)**: cerebellar (CBV), **(H)**: brainstem (BM). Solid lines depict the 50th centile, and dotted lines the 5th and 95th centiles. Red square (F): female fetuses; black triangle (M): male fetuses.

The tabular summaries of mean, SD, minimum, maximum, median, and 25th and 75th quartiles from the base data of TBV, GMV, SBV, ICV, e-CSFV, VV, CBV, and BM for fetuses between 19 and 37 GA are shown in Tables 1A–H. All volumetric measurements had significant positive correlations with GA and the quadratic lines for *CI*'s for each GA determined by the best regression fit for each structure are shown in Figure 3. Our measured volumes data were used to derive a best-fit formula, TBV = 0.45GA^2^-9.57GA + 47.41(R^2^ = 0.98); ICV = 0.46GA^2^ – 2.10GA – 69.30 (R^2^ = 0.98); GMV = 0.21GA^2^ – 7.60GA + 77.78 (R^2^ = 0.97); SBV = 0.17GA^2^ + 0.70GA – 58.48 (R^2^ = 0.97); e-CSFV = 0.46GA^2^ – 2.10GA – 69.30 (R^2^ = 0.98); VV = 0.03GA^2^ – 1.40GA + 18.99 (R^2^ = 0.57); CBV = 0.06GA^2^ – 2.37GA + 25.51 (R^2^ = 0.97); BM = 0.007GA^2^ – 0.46GA + 0.16 (R^2^ = 0.89). Measured mean ICV values at 19 weeks are significantly different from those previously reported (*p* < 0.05). When comparing the mean TBV values for each GA week as generated by the Jarvis' formula, our data were found to approximate the prediction at every GA week except weeks 22, 26, 29, and 30 (all *p* < 0.05; Table 2). The predicted mean TBV value generated by our formula (TBV = 0.45GA^2^ - 9.57GA + 47.41) were very similar at every GA week to values predicted by the Jarvis' formula, and the predicted TBV means derived from the previously reported formula were all within the 95% *CI* of the predicted means of this study (Table 3).

**Table 1:** The tabular summaries of min, max, mean, 25th, 50th, and 75th centiles of **(A)** TBV, **(B)** ICV, **(C)** GMV, **(D)** SBV, **(E)** e-CSFV, **(F)** VV, **(G)** CBV, and **(H)** BM.

			**A:TBV(ml)**				
**GA**	**Min**	**25th**	**50th**	**75th**	**Max**	**Mean**	**SD**
37	305.00	307.50	318.00	325.80	331.60	316.90	10.20
36	280.80	285.10	293.00	303.40	306.80	294.00	9.97
35	244.10	249.30	260.60	269.70	278.80	260.30	12.11
34	228.50	229.10	234.00	245.10	250.80	236.90	8.87
33	196.50	202.40	210.50	220.10	231.90	212.10	11.10
32	180.90	190.80	196.70	200.70	208.60	197.80	6.43
31	165.20	175.40	184.30	189.40	192.40	182.00	8.92
30	166.50	170.00	176.70	180.80	184.90	175.60	6.09
29	140.00	156.90	166.90	175.60	178.70	164.10	11.55
28	122.30	131.90	138.00	152.20	157.10	140.80	11.65
27	107.00	114.6	124.30	130.50	140.80	122.30	10.18
26	82.42	88.75	92.00	93.92	97.00	91.10	4.04
25	76.00	80.38	83.69	86.42	88.00	83.17	3.80
24	64.82	68.42	74.46	80.10	88.90	74.98	6.60
23	58.58	61.48	65.81	67.21	72.80	64.98	3.78
22	51.42	53.32	55.90	58.59	61.62	56.08	3.66
21	40.11	42.05	47.00	51.35	57.41	47.28	5.63
20	33.10	35.33	37.95	39.75	41.90	37.63	2.87
19	26.80	27.25	29.00	31.73	32.50	29.33	2.38
			**B:ICV (ml)**				
**GA**	**Min**	**25th**	**50th**	**75th**	**Max**	**Mean**	**SD**
37	480.00	480.20	493.30	502.80	505.50	491.80	11.47
36	448.80	449.60	463.60	468.50	470.00	460.00	9.74
35	384.50	410.40	432.40	443.30	451.10	426.70	23.28
34	367.50	376.40	387.50	398.30	402.70	386.60	12.09
33	330.60	333.70	345.50	354.20	358.00	344.50	10.24
32	308.90	321.50	330.20	336.30	340.40	328.80	9.25
31	283.60	296.00	308.50	312.30	324.00	304.70	12.19
30	278.90	287.70	306.50	317.00	319.60	302.50	15.51
29	256.00	266.70	274.40	289.00	301.00	277.50	13.94
28	225.60	234.10	246.10	260.60	269.70	246.80	15.27
27	187.50	196.90	212.80	235.50	253.40	215.50	21.33
26	157.60	167.50	176.00	183.00	189.00	175.40	9.45
25	143.70	149.60	158.30	167.00	173.10	158.50	9.53
24	122.20	128.70	139.50	147.50	160.10	141.50	12.11
23	112.50	118.70	123.50	135.60	140.60	125.70	9.34
22	94.00	98.64	106.90	119.60	128.40	109.30	11.90
21	79.26	80.78	86.00	91.36	95.60	86.40	5.65
20	73.50	74.99	76.89	78.23	80.00	76.74	2.13
19	65.70	66.53	69.75	72.38	73.00	69.55	3.05
			**C:GMV (ml)**				
**GA**	**Min**	**25th**	**50th**	**75th**	**Max**	**Mean**	**SD**
36	80.80	81.40	83.40	86.80	89.10	83.96	3.20
36	68.67	72.78	79.58	80.57	81.00	77.26	5.04
35	60.00	61.56	64.79	69.39	70.89	65.24	4.23
34	52.35	55.10	58.77	61.29	63.49	58.23	3.70
33	42.86	44.64	47.50	50.18	54.79	47.84	3.68
32	39.87	41.74	45.67	49.61	52.38	45.78	4.14
31	37.65	39.13	40.34	42.42	46.33	40.95	2.53
30	34.23	36.49	39.00	43.26	45.69	39.71	3.76
29	27.00	29.60	32.06	37.72	40.00	33.44	4.44
28	26.22	28.19	29.00	32.49	34.09	29.98	2.66
27	20.18	23.46	24.60	25.75	26.91	24.31	1.81
26	16.69	17.90	18.00	19.40	20.68	18.47	1.18
25	15.45	16.09	17.00	17.50	18.66	16.96	0.96
24	13.46	14.78	15.59	16.09	17.03	15.42	0.97
23	12.24	13.00	13.54	14.35	15.01	13.63	0.88
22	10.77	11.52	12.03	12.74	13.02	12.05	0.68
21	8.14	9.23	10.00	10.66	11.89	9.99	1.08
20	6.55	6.99	7.23	7.70	8.00	7.28	0.48
19	6.00	6.12	6.63	6.95	7.00	6.57	0.43
			**D:SBV (ml)**				
**GA**	**Min**	**25th**	**50th**	**75th**	**Max**	**Mean**	**SD**
37	194.50	196.90	210.40	215.60	220.40	207.10	10.27
36	185.20	186.30	190.00	201.90	207.30	193.30	8.88
35	165.80	167.30	176.10	182.00	186.30	175.50	7.95
34	153.10	156.10	160.70	165.90	176.70	162.10	7.39
33	138.50	142.60	148.50	156.00	167.80	150.00	9.27
32	119.10	131.90	135.20	140.40	152.70	137.70	8.89
31	114.60	124.40	130.10	135.20	139.30	129.30	7.60
30	112.50	120.50	125.90	130.60	133.10	124.90	6.29
29	102.10	110.3	125.00	128.80	139.90	120.80	11.43
28	75.39	90.15	99.42	111.33	115.81	99.43	12.54
27	78.99	82.77	89.91	99.20	107.40	91.02	9.20
26	57.46	66.33	67.93	69.23	71.80	67.15	3.62
25	54.20	59.63	61.87	64.58	66.53	61.39	3.93
24	43.96	50.99	54.96	59.01	67.84	55.35	5.97
23	42.56	44.29	48.37	49.67	56.00	47.59	3.72
22	37.11	38.88	40.95	43.08	45.96	40.96	2.68
21	25.96	30.39	34.22	38.64	42.30	34.40	5.11
20	24.44	25.68	28.34	29.74	31.20	27.93	2.30
19	18.88	19.05	20.18	22.72	23.36	20.65	1.98
			**E:e-CSFV (ml)**				
**GA**	**Min**	**25th**	**50th**	**75th**	**Max**	**Mean**	**SD**
36	152.90	156.00	161.00	178.70	192.00	166.10	15.17
36	135.30	148.70	162.60	166.20	166.20	158.50	13.08
35	138.00	149.30	165.00	178.50	186.10	164.10	17.51
34	134.00	150.20	161.30	171.70	182.10	160.40	16.00
33	96.56	115.80	132.10	140.80	150.10	128.00	16.53
32	112.00	120.90	130.10	138.40	145.60	129.60	10.68
31	92.73	114.30	123.00	127.00	130.80	119.50	11.20
30	90.00	105.50	128.80	138.30	147.20	124.10	18.73
29	73.30	98.46	107.00	137.40	139.10	110.70	20.53
28	89.17	95.01	98.22	113.20	116.60	103.10	10.13
27	64.20	73.72	87.17	103.80	131.50	90.38	21.36
26	66.74	73.86	84.22	87.42	93.22	81.57	8.24
25	54.55	61.74	74.59	83.18	88.48	72.38	11.33
24	43.36	55.07	63.36	75.44	88.27	63.76	12.21
23	44.22	53.94	57.72	65.25	68.09	57.23	7.42
22	33.00	39.81	47.74	62.15	64.28	50.17	11.28
21	25.52	34.68	37.90	42.36	44.99	37.60	5.85
20	36.02	36.47	37.08	37.96	39.46	37.32	1.09
19	36.80	37.05	38.35	39.05	39.10	38.15	1.07
			**F:VV (ml)**				
**GA**	**Min**	**25th**	**50th**	**75th**	**Max**	**Mean**	**SD**
37	8.00	8.25	9.00	9.40	9.50	8.86	0.61
36	6.66	6.83	7.80	8.05	8.10	7.51	0.64
35	3.04	4.84	6.32	6.99	7.84	5.94	1.64
34	3.00	4.00	5.25	6.13	7.13	5.11	1.42
33	2.14	3.50	4.13	5.24	6.20	4.34	1.24
32	1.69	2.53	3.46	4.55	5.53	3.50	1.22
31	1.99	2.34	3.15	3.90	5.00	3.20	0.95
30	1.58	2.00	2.82	3.17	4.00	2.70	0.72
29	2.00	2.00	2.24	3.79	4.00	2.73	0.85
28	1.78	2.47	2.75	3.41	4.83	2.98	0.89
27	1.15	2.32	3.00	3.69	4.00	2.90	0.92
26	1.86	2.00	2.34	3.60	4.14	2.74	0.86
25	1.50	2.43	2.85	3.74	4.34	2.97	0.82
24	1.13	2.08	2.56	3.76	4.29	2.73	0.93
23	1.70	2.64	3.32	4.00	4.60	3.29	0.84
22	2.07	218	3.20	3.51	4.64	3.05	0.79
21	1.00	1.17	1.50	1.84	2.34	1.53	0.43
20	1.00	1.03	1.77	2.51	3.00	1.80	0.77
19	1.60	1.65	1.95	2.63	2.80	2.08	0.53
			**G:CBV (ml)**				
**GA**	**Min**	**25th**	**50th**	**75th**	**Max**	**Mean**	**SD**
37	17.96	18.23	18.78	19.45	19.45	18.83	0.64
36	16.98	17.35	17.82	18.13	18.26	17.76	0.48
35	11.95	13.59	15.00	17.07	17.72	15.14	2.00
34	11.28	11.31	12.69	14.11	14.25	12.71	1.32
33	8.45	9.48	10.20	10.94	15.10	10.49	1.91
32	9.11	9.57	10.00	11.21	12.44	10.43	0.99
31	6.63	7.39	8.34	9.18	10.00	8.32	1.09
30	6.45	6.94	7.58	8.02	9.00	7.54	0.76
29	6.00	6.30	6.72	7.00	7.77	6.75	0.54
28	4.45	4.99	5.84	5.98	6.75	5.63	0.70
27	3.40	3.87	4.50	4.89	6.00	4.50	0.77
26	2.52	2.96	3.74	4.10	4.67	3.58	0.70
25	2.47	2.67	3.11	3.73	4.03	3.15	0.55
24	2.05	2.20	2.64	2.82	3.69	2.64	0.49
23	2.10	2.22	2.47	2.54	2.82	2.41	0.22
22	1.25	1.51	1.72	2.20	2.67	1.84	0.43
21	1.35	1.56	1.80	2.14	2.47	1.85	0.36
20	1.25	1.36	1.49	1.69	2.00	1.54	0.24
19	1.16	1.18	1.34	1.45	1.45	1.32	0.15
			**H:BM (ml)**				
**GA**	**Min**	**25th**	**50th**	**75th**	**Max**	**Mean**	**SD**
37	6.60	6.70	7.00	7.48	7.66	7.07	0.42
36	4.40	5.04	5.98	6.22	6.27	5.70	0.76
35	4.30	4.45	4.89	5.29	5.57	4.89	0.46
34	3.37	3.55	3.95	4.12	4.15	3.87	0.30
33	2.91	3.51	3.76	4.23	4.71	3.83	0.53
32	3.00	3.61	3.87	4.00	4.15	3.79	0.32
31	2.79	3.16	3.54	3.98	4.00	3.54	0.42
30	2.70	3.14	3.62	3.73	4.05	3.49	0.41
29	2.50	2.88	3.00	3.36	3.70	3.09	0.35
28	2.14	2.50	3.12	3.75	4.00	3.14	0.67
27	1.76	1.94	2.40	2.85	3.40	2.42	0.52
26	1.35	1.62	1.86	2.06	2.60	1.90	0.39
25	1.15	1.52	1.64	1.80	2.45	1.67	0.34
24	1.01	1.30	1.45	1.88	2.23	1.57	0.37
23	1.10	1.14	1.28	1.49	1.88	1.36	0.24
22	0.93	1.08	1.24	1.39	1.64	1.23	0.21
21	0.71	0.80	1.10	1.24	1.33	1.05	0.23
20	0.61	0.73	0.89	1.00	1.05	0.87	0.16
19	0.60	0.64	0.82	0.90	0.91	0.79	0.14

*(A): TBV, total brain volume; (B): ICV, intracranial cavity volume; (C): GMV, gray matter volume; (D): SBV, subcortical brain volume; VV, and (E): e-CSFV, extra-cerebrospinal fluid; (F) VV, lateral ventricles volume; (G): CBV, cerebellar volume; (H): BM, brainstem volume*.

**Table 2:** Total brain volume and ICV analysis compared with values reported by Jarvis' study.

**TBV Analysis**				**ICV Analysis**		
**GA**	**Mean**	**Mean difference** **(95% confidence)**	***P***	**Mean**	**Mean difference** **(95% confidence)**	***P***
36 (*n =* 5)	−3.81	(−20.12, 12.49)	0.59	−8.02	(−59.24, 43.20)	0.73
35 (*n =* 6)	5.90	(−11.95, 23.75)	0.46	−2.30	(−52.94, 48.33)	0.92
34 (*n =* 8)	6.40	(−6.01, 41.42)	0.23	10.21	(−28.68, 49.09)	0.58
33 (*n =* 9)	9.36	(−5.58, 18.81)	0.27	24.62	(−10.70, 59.94)	0.15
32 (*n =* 12)	5.00	(−4.63, 14.63)	0.28	12.59	(−15.21, 40.40)	0.35
31 (*n =* 11)	−1.10	(−12.90, 10.71)	0.84	8.86	(−23.45, 41.17)	0.56
30 (*n =* 10)	−13.41	(−21.56, −5.25)	0.004^*^	−16.73	(−49.30, 15.84)	0.29
29 (*n =* 11)	−19.45	(−34.50, −4.41)	0.02^*^	−19.30	(−48.38, 9.78)	0.18
28 (*n =* 9)	−12.77	(−28.37, 2.84)	0.10	−15.69	(−46.94, 15.55)	0.30
27 (*n =* 13)	−9.82	(−22.85, 3.21	0.13	−10.63	(−39.43, 18.17)	0.45
26 (*n =* 13)	6.87	(1.52, 12.22)	0.02^*^	4.03	(−16.48, 24.55)	0.68
25 (*n =* 12)	1.36	(−3.74, 6.46)	0.57	−3.28	(−22.97, 16.41)	0.73
24 (*n =* 15)	−2.88	(−11.25, 5.49)	0.48	−8.73	(−26.51, 9.06)	0.32
23 (*n =* 15)	−4.18	(−9.08, 0.71)	0.09	−13.74	(−13.00, 40.48)	0.27
22 (*n =* 13)	−5.62	(−9.90, −1.33)	0.01^*^	−14.70	(−30.81, 1.40)	0.07
21 (*n =* 9)	−6.05	(−13.89, 1.79)	0.12	−7.18	(−20.69, 6.33)	0.27
20 (*n =* 8)	−4.63	(−9.67, 0.42)	0.07	−11.04	(−22.61, −0.52)	0.06
19 (*n =* 4)	−3.43	(−11.37, 4.52)	0.32	−16.47	(−31.83, −1.11)	0.04^*^

* *Denotes statistical significance*.

**Table 3:** Total brain volume derived by our formula compared with predictive inference values calculated with formula by Jarvis et al.

**GA**	**Predicted Mean Value**	**Lower Predicted CI**	**Upper Predicted CI**	**Prediction by Jarvis' formula**
37	309.82	288.93	330.71	–
36	286.52	265.85	307.19	290.20
35	264.12	243.60	284.63	266.20
34	242.62	222.19	263.04	243.30
33	222.02	201.64	242.40	221.50
32	202.32	181.96	222.68	200.70
31	183.52	163.17	203.88	180.90
30	165.62	145.27	185.98	162.20
29	148.63	128.27	168.99	144.60
28	132.53	112.17	152.89	128.00
27	117.33	96.98	137.69	112.40
26	103.04	82.69	123.39	98.00
25	89.64	69.30	109.98	84.50
24	77.15	56.81	97.48	72.10
23	65.55	45.20	85.90	60.80
22	54.86	34.47	75.24	50.50
21	45.06	24.61	65.52	41.20
20	36.17	15.58	56.76	33.00
19	28.18	7.39	48.96	25.90

The relative growth rate of the volume of different intracranial structures is as follows: ICV: 10.87%; TBV: 13.22%; GMV: 14.15%; SBV: 12.81%; e – CSFV: 8.12%; VV: 8.05%; CBV: 14.77%; and BM: 12.18%.

### Effect of Sex

Male fetuses had slightly larger measurements compared with female fetuses in any intracranial structure of the 3D measurements expect for e-CSFV (male fetuses, 93.08; female fetuses, 93.31; *p* = 0.97), while the difference between sexes were not significant in ICV (male fetuses, 234.0; female fetuses, 226.0; *p* = 0.63), TBV (male fetuses, 137.2; female fetuses, 129.6; *p* = 0.50), GMV (male fetuses, 31.16; female fetuses, 29.11; *p* = 0.48), SBV (male fetuses, 96.95; female fetuses, 91.91; *p* = 0.50), CBV (male fetuses, 6.38; female fetuses, 6.01; *p* = 0.60), and BM (male fetuses, 2.72; female fetuses, 2.56; *p* = 0.47). The largest sex-related differences were significantly higher volumes in male fetuses for the lateral ventricles (male fetuses, 3.69; female fetuses, 3.08; *p* = 0.01).

## Discussion

Quantitative image analysis of the human brain *in utero* plays an important role in clinical decision-making and neuroscience investigation. With the advent of image post-processing technology and motion correction algorithms to obtain high-quality 3D images (Kim et al., 2010), it is now possible to improve the accuracy of manual segmentation of the fetal brain in the early and middle trimesters (Habas et al., 2010). We have presented normative data of the intracranial contents from a large cohort (*n* = 188) of control fetuses and individualized data on the regional fetal brain volumes (not all these structures were assessed by previous articles) between 19 and 37 GA. In addition, we found that the largest sex-related differences were significantly higher volumes in male fetuses for the VV.

In recent years, different studies have analyzed and reported changes in fetal brain volume (Jarvis et al., 2019; Cai et al., 2020; Dovjak et al., 2021), but the results are inconsistent and may depend on different measurement methods and whether or not fetal movement artifacts are processed. In addition, the number of fetuses in the cohorts studied varied widely in these studies, from the smaller cohort of 25 fetuses reported by Gholipour et al. (2017) to the largest cohort of 659 normal fetuses studied by Shi et al. (2020), which proposed an automated fetal brain analysis method, such as brain extraction, 3D volumetric reconstruction, atlas generation, and quantification of brain development. This method reduces the time required for manual editing following automatic segmentation to achieve such a surprising amount. While the data in most studies with large sample sizes are acquired from multi-institutions with multi-sequences (SSFSE and steady-state free precession, SSFP) on different scanners (different field strength and manufacturers). We are looking forward to using a single device and a single sequence to get a large sample of normative data of fetal intracranial structures, which would be more meaningful and perfect. So the accurate manual segmentations as prior knowledge could be used for the design and verification of the automatic volumetric segmentation method.

We made a like-to-like comparison of our total fetal brain volumes with those predicted by Jarvis et al., which used a large cohort (*n* = 200) of control fetuses and individualized data on the intracranial volumes between 18 and 37 GA (Jarvis et al., 2016, 2019). While a major strength of Jarvis' study is the inclusion of 200 fetuses across a wide GA range, they limited their measurements to total and regional brain structures without different tissue types (e.g., cortical gray matter and white matter). In an earlier publication (Jarvis et al., 2016), they reported on the TBV (only) from 132 of the cases reported in this manuscript along with a prediction equation. By substituting GA into this model, the difference between the actual and theoretical mean values of TBV can be analyzed to obtain a more accurate assessment of fetal brain development. Jarvis' formula measured an average TBV of 25.9 ml at 19 weeks gestation and 290.6 ml at 36 GA. In our study, the mean TBV of 188 fetuses was 29.33 ml at 19 weeks and 294 ml at 36 weeks. When comparing the mean TBV with the expected values generated by the Jarvis' formula for each GA week, our data were found to approximate the prediction at every GA week except weeks 22, 26, 29, and 30 (*p* < 0.05).

A number of reasons could explain this difference. First, the thickness of their MRI acquisitions ranged from 2 to 2.6 mm, whereas ours was 2 mm thick. Another source of bias is that the reconstructed volumetric images allowed us to develop supervised image segmentation techniques to improve the accuracy and ease of obtaining precise fetal brain volumetry. These may have introduced large errors in volume measurements, especially for small intracranial structures. The results generated by our formula were very similar to values predicted by the Jarvis' formula at every GA week. Therefore, using the formula by Jarvis' to predict expected small intracranial structures is based on extrapolation by the formula, which introduces potential error. So, our study reinforces the formula by Jarvis et al. but provides more robust total fetal brain volume measurements in the earlier second and third stages of pregnancy as a result of our larger study population.

From our results, the GMV followed a quadratic growth pattern, indicating accelerated growth at this stage of development, as demonstrated by the progressing growth velocity in brain volume in the later middle and third trimesters. The GMV increased at a relative growth rate of 14.15% per week in our study. This is consistent with a previous study (Kyriakopoulou et al., 2017) performed on 127 normal fetuses at 21–38 gestational weeks (14.78%). Although the overall growth rate of GMV and SBV is not very different throughout pregnancy, the growth trajectory and proportion of cerebral volume of GMV and SBV between 18 and 37 weeks are different. We found that subcortical white matter is a major contributor to fetal brain volume development during the middle and later trimesters of pregnancy, reaching the peak between 29 and 30 weeks of gestation. Our results showed that the proportion of cerebral cortex to the fetal total brain volume in the late trimester increased significantly with the increase of gestation.

Our results indicated that the relative growth rates varied between structures with the CBV (14.77%), which showed the fastest growth per week followed by the cortex and the supratentorial brain tissue, while the growth of the lateral ventricles was the slowest (8.05%). During the second and third trimesters of pregnancy, the cerebellum undergoes extensive proliferation and migration of external granulosa cells, and the formation of the internal granulosa layer, which are the basis for significant increase in CBV (Griffiths et al., 2004; Bolduc et al., 2012). Reductions in total cerebellum and local volume in infants with microcephaly are associated with delays in cognition, motor function, and social-affective disorders.

The size of lateral ventricles can be used to predict fetal nervous system dysfunction (Carta et al., 2018; Fox et al., 2018). Therefore, the accurate measurement of bilateral ventricles volume is crucial to the diagnosis of lateral ventricle enlargement, and ventriculomegaly is an indicator of fetal brain development abnormalities. In our study, we found that male fetuses had significantly larger VV compared with female fetuses. This result is supported by extensive US data, which consistently report that the standard 2-D indicators of ventricular diameter are larger in male fetuses (Salomon et al., 2007). This result is very critical, as ventriculomegaly is frequently encountered at fetal MRI. So the difference in the VV between fetuses of different sexes suggests that this variable should be considered in the assessment of ventriculomegaly.

There are some limitations to this study. First, the number of fetal brains in our cohort for 3D construction is still limited, because the poor imaging quality caused by motion artifacts, causing failures of fetal brain super-resolution reconstruction. We acknowledge that we did not have successful neurodevelopment outcomes for all of the children who had been studied as normal fetuses in our cohort. However, previous research (Griffiths et al., 2017) has shown that the false positive and negative rates for detecting abnormalities by prenatal MR are very low. In the future, we hope to optimize the reconstructed algorithm and segmental process with the eventual aim to provide accurate automatic segmentation.

The normative values of fetal intracranial structures across a broad range of gestations with associated prediction limits could potentially be used as a reference tool in prenatal counseling. Volumetric growth of the fetal brain follows a complex trajectory that is dependent on structure, GA, and sex. Therefore, we propose preferential use of these measured mean values over formula-derived predictions in clinical counseling for fetuses with GA in the early second and third stages of pregnancy.

## Data Availability Statement

The original contributions presented in the study are included in the article, further inquiries can be directed to the corresponding author.

## Ethics Statement

The studies involving human participants were reviewed and approved by Shanghai Children's Medical Center. The patients/participants provided their written informed consent to participate in this study.

## Author Contributions

J-YR performed experiments, analyzed the data, and drafted the manuscript. S-ZD and MZ designed this study, analyzed the fetal MRI data, and revised the manuscript. G-HW, Y-DG, and FJ designed the cohort and following up. All authors read and approved the final manuscript.

## Funding

This work was supported by the National Natural Science Foundation of China (nos. 81971582 and 81571628), the Natural Science Foundation of Shanghai (no. 19ZR1476700), the Shanghai Pujiang Program (no. 2019PJD030), the Collaborative Innovation Program of Shanghai Municipal Health Commission (no. 2020CXJQ01), the Shanghai Jiao Tong University School of Child Developing Brian Research Center Construction Funds, and the Shanghai Jiao Tong University School of Medicine (Innovation Team on Pediatric Research Funds).

## Conflict of Interest

The authors declare that the research was conducted in the absence of any commercial or financial relationships that could be construed as a potential conflict of interest.

## Publisher's Note

All claims expressed in this article are solely those of the authors and do not necessarily represent those of their affiliated organizations, or those of the publisher, the editors and the reviewers. Any product that may be evaluated in this article, or claim that may be made by its manufacturer, is not guaranteed or endorsed by the publisher.

## Abbreviations

GA: Gestational age
2D: Two-dimensional
3D: Three-dimensional
ICV: Intracranial cavity volume
TBV: Total brain volume
VV: Lateral ventricles volume
CBV: Cerebellar volume
GMV: gray matter volume
SBV: subcortical brain volume
BM: Brainstem volume
E-CSFV: Extra-cerebrospinal fluid.
